# 125例自身免疫性溶血性贫血患者的治疗实践与结局：单中心回顾性研究

**DOI:** 10.3760/cma.j.cn121090-20251209-00582

**Published:** 2026-03

**Authors:** 斐 杨, 若难 李, 乐乐 张, 珍 高, 虹 潘, 林珠 田, 昱灿 沈, 文燕 王, 伟望 李, 婧余 赵, 丽云 李, 潇 于, 静 徐, 纯 许, 燕杰 刘, 为为 王, 哲湘 匡, 能 聂, 星鑫 李, 建平 李, 金波 黄, 馨 赵, 静 张, 力维 方, 悦晨 罗, 均 施

**Affiliations:** 1 中国医学科学院血液病医院（中国医学科学院血液学研究所），血液与健康全国重点实验室，国家血液系统疾病临床医学研究中心，细胞生态海河实验室，天津 300020 State Key Laboratory of Experimental Hematology, National Clinical Research Center for Blood Diseases, Haihe Laboratory of Cell Ecosystem, Institute of Hematology & Blood Diseases Hospital, Chinese Academy of Medical Sciences & Peking Union Medical College, Tianjin 300020, China; 2 天津医学健康研究院，天津 301600 Tianjin Institutes of Health Science, Tianjin 301600, China

**Keywords:** 自身免疫性溶血性贫血, 回顾性研究, 治疗结局, 靶向治疗, Autoimmune hemolytic anemia, Retrospective study, Treatment outcomes, Targeted therapy

## Abstract

**目的:**

探索单中心回顾性队列中自身免疫性溶血性贫血（AIHA）患者临床特征、治疗模式及结局。

**方法:**

回顾性分析2019年7月至2025年2月中国医学科学院血液病医院（中国医学科学院血液学研究所）收治的125例AIHA患者的临床资料，分析其临床特征、治疗模式及结局。

**结果:**

糖皮质激素单药是最常用的一线治疗方案，而78.2％（97/124）的患者在病程中接受过糖皮质激素联合利妥昔单抗治疗。75例患者接受一线糖皮质激素单药治疗，总体反应率（ORR）为61.3％，持续缓解率仅10.7％；25例接受一线糖皮质激素联合利妥昔单抗治疗，ORR为88.0％，持续缓解率为60.0％。中位治疗线数为2（1～4）线，约三分之一患者需接受三线及以上治疗。27例（21.6％）复发/难治患者在临床研究背景下接受新型靶向药物探索性用药，其治疗线数显著高于未接受者［4（2～12）线对2（1～6）线，*P*<0.001］，治疗后44.4％实现缓解，40.7％ HGB恢复正常。并发症方面，8例（6.4％）发生静脉血栓事件，36例（28.8％）发生感染，发生感染患者治疗线数显著高于无感染患者（*P*<0.001）。

**结论:**

AIHA治疗模式上具有高度异质性，糖皮质激素单药仍是最常用的一线治疗方案，但持续缓解能力有限。糖皮质激素联合利妥昔单抗治疗显示较高的ORR与持续缓解率。病程中发生感染患者治疗线数显著增加，提示临床实践中需重视感染的预防与监测。

## Abstract (en)

**Objective:**

To explore the clinical characteristics, treatment patterns, and outcomes of patients with autoimmune hemolytic anemia（AIHA）in a single-center retrospective cohort.

**Methods:**

Clinical data of 125 patients with AIHA admitted to the Blood Diseases Hospital, Chinese Academy of Medical Sciences, between July 2019 and February 2025, were retrospectively analyzed to investigate their clinical characteristics, treatment patterns, and outcomes.

**Results:**

Glucocorticoid monotherapy was the most commonly administered first-line treatment, whereas 78.2％ of the patients received glucocorticoids in combination with rituximab during the disease course. The overall response rate（ORR）and the sustained remission rate were 61.3％ and 10.7％ in 75 patients receiving glucocorticoid monotherapy as first-line treatment and 88.0％ and 60.0％ in 25 patients treated with glucocorticoids plus rituximab, respectively. The median number of treatment lines was two, with approximately one-third of the patients requiring three or more lines of therapy. In the context of clinical research, 27（21.6％）patients with relapsed/refractory disease received exploratory treatment with novel targeted agents, with a significantly higher median number of treatment lines compared with those who did not［4（2–12）*vs* 2（1–6）, *P*<0.001］. After treatment, 44.4％ of patients achieved remission, whereas 40.7％ of patients had their HGB levels normalized. Regarding complications, venous thromboembolism occurred in 8 patients（6.4％）. Further, 36 patients（28.8％）developed infections, with a significantly higher number of treatment lines in the infection group than in the noninfection group（*P*<0.001）.

**Conclusion:**

The therapeutic approaches for AIHA demonstrate remarkable heterogeneity. Glucocorticoid monotherapy remains the most commonly administered first-line treatment; however, its ability to maintain sustained remission is limited. Glucocorticoid treatment in combination with rituximab demonstrated a higher ORR and sustained remission rate. Patients who developed infections during the disease course required significantly more lines of therapy, emphasizing the importance of infection prevention and surveillance in clinical practice.

自身免疫性溶血性贫血（AIHA）是一种由红细胞自身抗体和（或）补体吸附于红细胞表面，导致红细胞破坏加速，超出骨髓代偿能力而引起的溶血性疾病。AIHA的发病率为0.8～3.0/10万人年，患病率为17/10万人年[1]。AIHA是一组异质性疾病，根据是否存在明确的病因，分为原发性与继发性；根据自身抗体结合红细胞所需要的最适温度分为温抗体型、冷抗体型及混合型，各类型AIHA在临床特征上存在显著差异。糖皮质激素是AIHA的一线治疗基石，但只有20％～30％的患者在糖皮质激素减量/停药后可实现持续缓解[2]，利妥昔单抗的应用显著改善了治疗结局，但仍有部分患者表现为复发/难治[3]。近年来，有多种新型靶向药物用于AIHA的治疗，为复发/难治患者再次诱导缓解提供了可能。当前，大多数AIHA的治疗推荐仍停留在专家共识层面，其推荐意见的修订与临床实践的规范，均亟需高质量的真实世界数据支持。因此，本研究基于回顾性临床数据，系统评估AIHA患者在真实世界中的治疗模式与临床结局，旨在明确当前治疗路径的实际应用现状，以期为规范临床决策提供循证依据。

## 病例与方法

1. 病例资料：本研究为单中心回顾性队列研究，连续收集2019年7月至2025年2月在中国医学科学院血液病医院再生医学诊疗中心诊断为AIHA患者的病例。诊断参考《自身免疫性溶血性贫血诊断与治疗中国专家共识（2017年版）》[4]，部分AIHA患者合并免疫性血小板减少，诊断符合Evans综合征定义[5]。本研究未排除继发性AIHA。入组患者出院后至少完成1次随访评估，未获得任何随访资料的患者不纳入研究。起病时临床特征（包括HGB水平、血清学分型等）主要依据患者首次出现溶血相关症状时的本院病历记录或外院完整病历资料获得。随访截至2025年5月31日，主要通过门诊复诊记录、住院病历及电话进行随访。

2. 疗效评价标准[6]：完全缓解（CR）：HGB≥110 g/L（女性）或HGB≥120 g/L（男性），溶血相关实验室指标［网织红细胞绝对值（Ret）、乳酸脱氢酶（LDH）、胆红素］均正常；部分缓解（PR）：HGB ≥ 100 g/L或较基线值增长≥20 g/L，且脱离红细胞输注≥7 d；总有效率（ORR）为CR率与PR率之和。

3. 统计学处理：应用SPSS 27.0统计软件进行数据分析。呈非正态分布的计量资料用中位数（范围）表示，计数资料用例数（构成比）表示。两组间计量资料的比较采用Mann-Whitney *U*检验；多组间计量资料的整体比较采用Kruskal-Wallis *H*检验，当差异有统计学意义时，进一步采用事后两两比较（Bonferroni法校正显著性水平）。*P*<0.05为差异具有统计学意义。

## 结果

一、临床和血清学特征

本研究共纳入125例AIHA患者，基线特征见表1。其中，男42例，女83例，初诊中位年龄45（5～84）岁。血清学类型：温抗体型94例（75.2％），混合型16例（12.8％），冷抗体型7例（5.6％），直接抗人球蛋白试验（DAT）阴性8例（6.4％）。此外，19例患者合并免疫性血小板减少，明确诊断为Evans综合征。

**表1:** 125例自身免疫性溶血性贫血（AIHA）患者临床特征

临床特征	数值
性别（例，男/女）	42/83
初诊年龄［岁，*M*（范围）］	45（5～84）
初诊年龄分组［例（％）］	
<18岁	13（10.4）
18～45岁	53（42.4）
46～65岁	42（33.6）
>65岁	17（13.6）
AIHA血清学类型［例（％）］	
温抗体型	94（75.2）
冷抗体型	7（5.6）
混合型	16（12.8）
DAT阴性	8（6.4）
起病时HGB［g/L，*M*（范围）］^a^	60（20～135）
起病时LDH［U/L，*M*（范围）］^b^	489（144～3 093）
起病时TBIL［µmol/L，*M*（范围）］^c^	58.1（9.6～252.9）
起病时Ret［×10^9^/L，*M*（范围）］^d^	257.3（10.4～576.8）
治疗线数［*M*（范围）］	2（1～14）
随访时间［月，*M*（范围）］	13（1.5～72）

**注** DAT：直接抗人球蛋白试验；LDH：乳酸脱氢酶；TBIL：总胆红素；Ret：网织红细胞绝对值。^a^116例患者可获得起病时HGB数据；^b^65例患者可获得起病时LDH数据；^c^87例患者可获得起病时TBIL数据；^d^45例患者可获得起病时Ret数据

116例患者可获得起病时HGB数据，中位HGB为60（20～135）g/L。按贫血严重程度分层，59例（50.8％）HGB≤60 g/L，32例（27.6％）60 g/L<HGB≤80 g/L，21例（18.1％）80 g/L<HGB≤100 g/L，4例（3.4％）HGB>100 g/L。组间比较显示，不同血清学类型AIHA患者之间的起病时HGB水平差异有统计学意义（*P*＝0.012）；事后两两比较发现，温抗体型及混合型AIHA患者起病时HGB水平显著低于冷抗体型患者（*P*值分别为0.016、0.012）（图1A）。值得注意的是，在具备起病时HGB数据的混合型AIHA患者中，高达73.3％（11/15）起病即表现为极重度贫血（HGB≤60 g/L）。

**图1:**
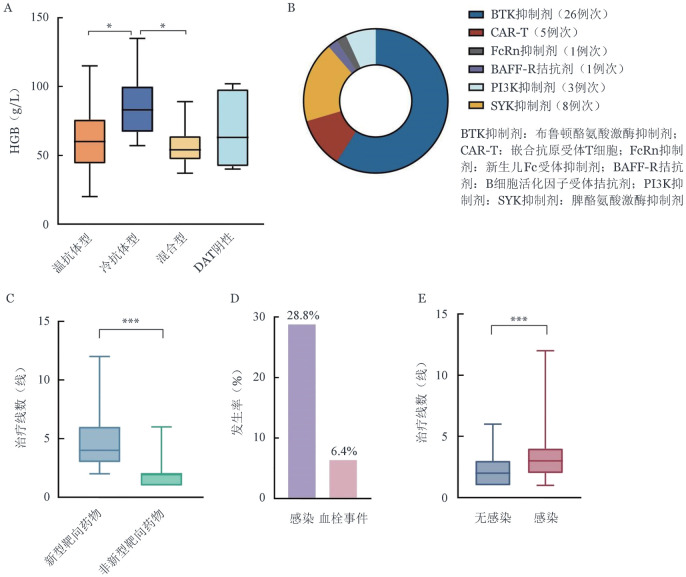
自身免疫性溶血性贫血（AIHA）患者的临床特征、靶向治疗模式与并发症分析（*P<0.05、***P<0.001） **A** 不同血清学分型AIHA患者起病时HGB水平比较［温抗体型87例，冷抗体型7例，混合型15例，直接抗人球蛋白试验（DAT）阴性7例］；**B** 接受新型靶向药物治疗患者的具体用药种类分布；**C** 新型靶向药物组（27例）与非新型靶向药物组（97例）患者的治疗线数比较；**D** AIHA患者总体感染（36例）与血栓事件（8例）的发生率；**E** 发生感染（36例）与未发生感染（89例）患者的治疗线数比较

由于部分回顾性数据缺失，仅对具备有效资料的患者进行溶血指标统计：65例具备起病时LDH数据，中位LDH为489（144～3 093）U/L；87例具备起病时总胆红素（TBIL）数据，中位TBIL为58.1（9.6～252.9）µmol/L；45例具备起病时Ret数据，中位Ret为257.3（10.4～576.8）×10^9^/L。考虑到上述指标的可用病例数较少，按血清学分型进一步拆分后亚组样本量不足，为避免统计偏倚，未对不同分型间的LDH、TBIL及Ret水平进行比较分析。

并发症方面，8例（6.4％）患者发生血栓事件，其中6例为温抗体型，1例为冷抗体型，1例为混合型。由于事件数有限，未对不同分型间的血栓风险进行比较分析。所有血栓均为静脉血栓事件，其中1例为经外周静脉置入中心静脉导管（PICC）相关血栓，1例为股静脉置管相关血栓，另1例表现为下肢静脉血栓合并肺栓塞。

二、治疗模式与生存状态

排除1例仅接受中药治疗的患者，对其余124例AIHA患者的治疗模式进行分析。16例患者仅接受过糖皮质激素单药治疗，12例患者仅接受过糖皮质激素联合免疫抑制剂治疗，78.2％（97/124）的患者在病程中接受过糖皮质激素联合利妥昔单抗治疗，另有3例患者接受脾切除术（均为温抗体型AIHA）。

就一线治疗而言，本队列中共有75例患者接受糖皮质激素单药治疗，ORR为61.3％（46/75），但仅10.7％（8/75）能在糖皮质激素减量/停药后实现HGB持续缓解。对于一线糖皮质激素单药治疗无效或复发的67例患者中，67.1％（45/67）通过免疫抑制剂或利妥昔单抗再次获得缓解。25例患者一线接受糖皮质激素联合利妥昔单抗治疗，ORR为88.0％（22/25），且60.0％（15/25）实现持续缓解。鉴于本回顾性队列未对不同干预组的基线特征进行匹配校正，上述结果仅作描述性呈现，未进行方案间疗效的直接统计学比较。

在7例冷抗体型患者中，1例仅初期短暂应用糖皮质激素，HGB持续正常；3例一线糖皮质激素单药治疗未获得持续缓解，后续经利妥昔单抗治疗后均再次获得缓解；余3例一线接受利妥昔单抗治疗，随访期末1例维持PR，2例在起效1年内复发。

患者病程中接受治疗的中位线数为2（1～14）线，38例（30.6％）仅接受一线治疗，40例（32.3％）接受二线治疗，22例（17.7％）接受三线治疗，24例（19.4％）接受四线及以上治疗（表2）。进一步按血清学类型分层，其中87.5％（14/16）的混合型AIHA患者需接受二线及以上治疗。不同起病时HGB水平患者的治疗线数分布如表3所示，起病时贫血程度较重的患者中，仅接受一线治疗的比例相对较低（29.3％对40.0％），而接受四线及以上治疗的比例相对较高（24.1％对16.0％）。

**表2:** 不同自身免疫性溶血性贫血（AIHA）血清学类型患者的治疗线数分布［例（％）］

AIHA血清学类型	例数	治疗线数			
		一线	二线	三线	四线及以上
温抗体型	93	30（32.3）	24（25.8）	20（21.5）	19（20.4）
混合型	16	2（12.5）	10（62.5）	1（6.3）	3（18.7）
冷凝集素病	7	3（42.9）	2（28.6）	1（14.3）	1（14.3）
DAT阴性	8	3（37.5）	4（50.0）	0	1（12.5）

**注** DAT：直接抗人球蛋白试验

**表3:** 起病时不同HGB水平自身免疫性溶血性贫血（AIHA）患者的治疗线数分布［例（％）］

起病时HGB	例数	治疗线数			
		一线	二线	三线	四线及以上
≤60 g/L	58	17（29.3）	19（32.8）	8（13.8）	14（24.1）
61～80 g/L	32	8（25.0）	12（37.5）	6（18.8）	6（18.8）
>80 g/L	25	10（40.0）	7（28.0）	4（16.0）	4（16.0）

27例（21.6％）患者在传统治疗无效的情况下，在临床研究背景下接受新型靶向药物探索性用药。具体用药构成见图1B：布鲁顿酪氨酸激酶（BTK）抑制剂26例次、脾酪氨酸激酶（SYK）抑制剂8例次、嵌合抗原受体T细胞（CAR-T）5例次、磷脂酰肌醇3-激酶（PI3K）抑制剂3例次、B细胞活化因子受体（BAFF-R）拮抗剂及新生儿Fc受体（FcRn）抑制剂各1例次。接受新型靶向药物治疗的患者的治疗线数显著高于未接受新型靶向药物治疗组［4（2～12）线对2（1～6）线，*P*<0.001］（图1C）。新型靶向药物治疗后，27例患者中12例（44.4％）实现缓解，11例（40.7％）HGB恢复正常。

中位随访时间13（1.5～72）个月，124例患者整体结局如下：71.0％（88/124）患者HGB恢复至正常范围，其中52.4％（65/124）在末次随访时已完全停药。另有27.4％（34/124）患者HGB未恢复至正常，其中15.3％（19/124）患者的HGB<100 g/L，14.5％（18/124）患者处于复发/难治状态。中位随访时间13（1.5～72）个月，截至随访期末，本研究整体AIHA队列的结局如下：88例（71.0％，88/124）患者HGB恢复至正常范围，其中有65例（52.4％，65/124）患者在末次随访时已停用治疗。34例（27.4％，34/124）患者HGB未恢复至正常，其中19例（15.3％，19/124）患者的HGB<100 g/L，18例（14.5％，18/124）患者处于复发/难治状态。临床试验为复发/难治患者提供了进一步的治疗选择，但5例患者已经接受过新型靶向药物治疗：其中2例温抗体型AIHA患者在SYK抑制剂治疗后曾获得CR但后续复发，1例温抗体型AIHA患者BTK抑制剂治疗无效，另2例（1例温抗体型、1例混合型）在BCMA-CD19双靶CAR-T治疗后亦未获应答，提示此部分患者处于多重难治状态，对传统及新型靶向治疗均反应不佳，亟需新靶点干预。在个体结局方面，1例冷凝集素病患者HGB始终维持正常；另1例84岁女性AIHA患者后期进展为骨髓造血功能衰竭。

在全部125例AIHA患者中，共有36例（28.8％）在病程中发生过感染事件（图1D）。病程中感染组患者的中位治疗线数为3（1～12）线，高于无感染组的2（1～9）线，差异有统计学意义（*P*<0.001）（图1E）。

## 讨论

相较于血液系统恶性疾病，AIHA长期以来被视为一种相对容易治疗、临床影响较小的良性疾病。然而这一“良性”标签极易导致其在临床实践中被低估，使医患双方忽视其临床特征及治疗模式高度异质性、慢病程、易出现感染和血栓事件的特点，从而陷入对AIHA认知的误区[7]。本研究回顾性分析了125例AIHA患者的临床资料，系统评估了其血清学分型、疾病严重程度、治疗模式与生存状态以及并发症（感染和血栓事件）的发生情况，进一步揭示了AIHA异质性显著且管理复杂的临床现实。

AIHA在血清学类型及起病HGB水平上存在异质性。本研究提示，温抗体型为最常见血清学类型（占75.2％），混合型虽不常见，但临床表现更为凶险，73.3％的混合型患者起病时HGB ≤ 60 g/L，与既往研究基本一致[1]。本队列中冷抗体型AIHA有7例，均为冷凝集素病患者，其HGB水平显著高于温抗体型与混合型患者，可能与其IgM介导的肝脏主导溶血机制及发病缓慢相关[1]。

本研究显示糖皮质激素仍是AIHA患者中应用最为广泛的一线治疗药物。然而，一线糖皮质激素单药治疗的持续缓解率仅为10.7％，低于既往文献中报道的30％[8]，可能与本队列中约50％的患者在起病时即表现为极重度贫血，以及糖皮质激素减量过早、过快有关。近年来，已有指南将糖皮质激素联合利妥昔单抗推荐为AIHA的一线治疗策略，尤其适用于重度贫血患者[9]。尽管本研究中仅有部分患者一线接受该联合方案，但仍有78.2％的患者在整个病程中接受过糖皮质激素联合利妥昔单抗治疗，提示该治疗模式在真实世界临床实践中被广泛采用，并成为常用的治疗策略。在本研究中，接受糖皮质激素联合利妥昔单抗作为一线治疗的患者，60.0％可获得持续缓解。鉴于不同一线治疗方案间患者的基线特征未进行分层或校正，本文未对不同治疗方案间的疗效优劣进行直接比较。但既往随机对照研究已证实，在温抗体型AIHA中，糖皮质激素联合利妥昔单抗作为一线治疗可提高缓解率并延长无复发生存，且未明显增加不良反应[10]–[11]。对于冷抗体型患者，现行指南并不推荐糖皮质激素作为主要治疗手段，但在本研究中，糖皮质激素仍被较为频繁地使用，提示真实世界临床实践与指南推荐之间仍存在一定差距。在多线常规治疗无效后，40.7％（11/27）通过参加临床试验血象恢复正常，提示新型靶向药物在复发/难治AIHA中具有突破性潜力。但仍有5例多重难治患者，对于此类患者来说，仍应优先考虑临床试验入组。Zhang等[12]首次报道了2例在接受自体CD19 CAR-T细胞治疗后再次复发的多线治疗失败的AIHA患者，通过靶向BCMA的双特异性T细胞衔接器治疗，13～21 d HGB即可恢复正常，溶血标志物明显下降，且治疗后外周血B细胞和游离轻链在第2周迅速耗竭，骨髓浆细胞和B细胞在第12周显著清除，提示BCMA靶点对多重难治患者仍有效，值得优先纳入前瞻试验。

患者中位治疗线数为2线，约三分之一患者需接受三线及以上治疗，提示存在一定比例的复发/难治病例。87.5％的混合型AIHA患者需接受二线及以上治疗，进一步印证该亚型治疗反应较差。随着起病时HGB水平升高，治疗线数趋于减少，提示疾病起病时的贫血程度可能与后续治疗复杂度存在一定关联趋势。此外，接受新型靶向药物治疗组中位治疗线数高达4线，表明新药试验已成为多线失败后的挽救通道。近年来，多种靶向药物在AIHA治疗中展现出良好前景（如BTK抑制剂[13]–[16]、SYK抑制剂[17]、PI3K抑制剂、蛋白酶体抑制剂[18]及哺乳动物雷帕霉素靶蛋白抑制剂[19]等）。Li等[20]研究结果证明，CD19 CAR-T在难治性AIHA中可实现快速、长期的持续无药物缓解，且安全性可控，这一发现不仅验证了CAR-T疗法在AIHA中的治疗潜力，也为无药物缓解导向的新治疗方式奠定了理论与实践基础。

在本研究中，仅有3例（2.9％）温抗体型AIHA患者接受了脾切除术，提示临床实践中脾切除使用率显著下降。这一趋势与利妥昔单抗的广泛应用，医师对脾切除术后严重感染、血栓栓塞等并发症的重视密切相关。脾切除术作为一种侵袭性治疗方式，目前更多被保留用于激素及利妥昔单抗治疗失败后的个体化选择[21]。

一方面，AIHA患者由于自身免疫紊乱、糖皮质激素及免疫抑制剂的使用，感染发生率明显增加，本研究感染的发生率为28.8％。另一方面，感染本身亦可能通过诱发免疫紊乱、触发疾病复发，从而使患者进入更复杂的治疗过程。本研究显示，发生感染的患者其治疗线数显著高于未发生感染者，提示在AIHA的长期管理中，感染与治疗复杂度往往相互交织。既往研究表明感染是AIHA死亡的重要危险因素（*HR*＝11.47）[22]。因此，加强感染的预防、早期识别与规范处理，可能有助于优化AIHA的整体治疗过程。临床实践中，应加强患者宣教，指导患者发生感染时及时至医院就诊，同时对于高危患者（复发/难治、长期使用免疫抑制剂、脾切除患者）采取积极的预防感染措施（如：乙型肝炎病毒、丙型肝炎病毒等状态筛查，脾切除患者预防性疫苗接种）[23]。本研究中6.4％的患者发生血栓事件，低于既往文献报道的11％～20％[22]–[24]，这一差异可能源于以下原因：其一，本研究为单中心回顾性研究，部分无症状或轻度血栓事件可能因缺乏系统性筛查而被漏诊；其二，研究中位随访时间仅13个月，较短的随访时间可能低估了血栓的累积风险。未来可通过多中心、前瞻性研究准确评估不同亚型AIHA患者的血栓风险。本研究报道的8例静脉血栓事件中，2例（25.0％）与静脉置管相关。AIHA患者红细胞被破坏后释放的血红素可降低一氧化氮的生物利用度，诱导氧化应激反应并激活炎症通路，促发高凝状态[25]–[27]。此外，导管植入可能导致局部血流受阻，内皮细胞受损及局部高凝状态，从而进一步增加血栓形成风险。因此，在临床管理过程中，应加强对住院患者静脉通路管理，并动态评估其血栓风险。对于需长期置管的患者，是否采取预防性抗凝措施，应结合个体化风险评估综合权衡。

综上所述，本研究基于单中心回顾性队列数据，揭示了AIHA患者在临床特征、治疗模式及生存状态的多样性，同时勾勒出了AIHA的治疗模式由糖皮质激素单药向应用新型靶向药物并追求长期停药缓解的方向演进。未来应加强疾病分层与风险预测研究，加强高质量临床研究平台建设，为制定精准化、个体化治疗路径奠定基础，同时加快推进CAR-T、双特异性抗体等创新方案的前瞻性试验，让无药物缓解从个案走向常规，把复发/难治AIHA真正送入可治愈的精准治疗时代。本研究亦存在一定局限性，首先为单中心回顾性研究，可能存在选择偏倚；其次样本量较小，需扩大样本量，进一步推动AIHA患者的风险分层和个体化治疗路径的发展。
